# Paraganglioma Presenting as a Nasal Septal Mass: Case Report and Literature Review

**DOI:** 10.1155/2018/1413960

**Published:** 2018-12-06

**Authors:** James H. Kim, Nathan Tu, Bozena Barbara Wrobel

**Affiliations:** ^1^Keck School of Medicine of the University of Southern California, Los Angeles, CA, USA; ^2^University of Southern California LAC+USC Medical Center Department of Otolaryngology, Los Angeles, CA, USA; ^3^Associate Professor of Clinical Otolaryngology Head and Neck Surgery, Keck School of Medicine, Los Angeles, CA, USA

## Abstract

**Objectives:**

To describe a rare case of a paraganglioma arising from the nasal septum and review the diagnosis and management of paragangliomas in the nasal cavity and paranasal sinuses.

**Methods:**

We present a case of a 70-year-old female presenting with persistent nasal congestion and obstruction. Nasal endoscopy revealed a posterior septal mass approaching the sphenoid sinuses and partially obstructing the nasopharynx. A biopsy of the mass was taken, and histologic analysis confirmed a diagnosis of paraganglioma.

**Results:**

The patient underwent an endoscopic resection of the tumor. There has been no evidence of disease recurrence after 3 months of follow‐up.

**Conclusions:**

Paragangliomas arising from the nasal septum are exceedingly rare, but should be considered in the differential diagnosis in patients presenting with nasal septal masses. These tumors are typically benign, although few cases of malignant sinonasal paragangliomas have been reported. Treatment requires surgical excision with close follow-up as several cases of tumor recurrence have been reported.

## 1. Introduction

Paragangliomas are rare, slow-growing neuroendocrine tumors arising from cells of neural crest origin. They typically originate from the adrenal gland, but occur extra-adrenally in 5–10% of cases [1]. In the head and neck region, paragangliomas make up only 0.6% of all tumors, most commonly in the carotid body and jugular-tympanic regions [1–3]. Though rare, there are few reported cases of paragangliomas arising in the nasal cavity and paranasal sinuses [4–8]. To our knowledge, there has only been one previously reported case of a paraganglioma originating from the nasal septum [8].

## 2. Case Report

A 70-year-old female complaining of persistent nasal congestion and obstruction presented to our clinic for evaluation. She denied any headache or epistaxis. Nasal endoscopy was performed which showed a posterior septal mass approaching the sphenoid sinuses bilaterally and partially obstructing the view of the nasopharynx. The overlying mucosa was intact except for a small area superiorly which showed a soft granulomatous mass protruding into the left nasal cavity.

The patient was taken to the operating room for septoplasty with biopsy of the mass at an outside institution. Microscopic examination of the biopsy specimen demonstrated clusters of epithelioid-appearing cells separated by bands of fibrillary stroma. The epithelioid cells were noted to have abundant amphophilic cytoplasm, uniform, rounded nuclei with “salt and pepper” chromatin, and small nucleoli. No mitotic activity, invasion, necrosis, or calcification was seen. Immunohistochemical staining demonstrated positivity for neuron-specific enolase (NSE), chromogranin A, synaptophysin, and CD56 cell markers within the epithelioid cells. Fibrillary cells were positive for NSE, chromogranin A, S-100, glial fibrillary acid protein (GFAP), and CD56 cell markers. Based on the histological appearance and immunohistochemical staining, a diagnosis of paraganglioma was made.

**Figure 1:**
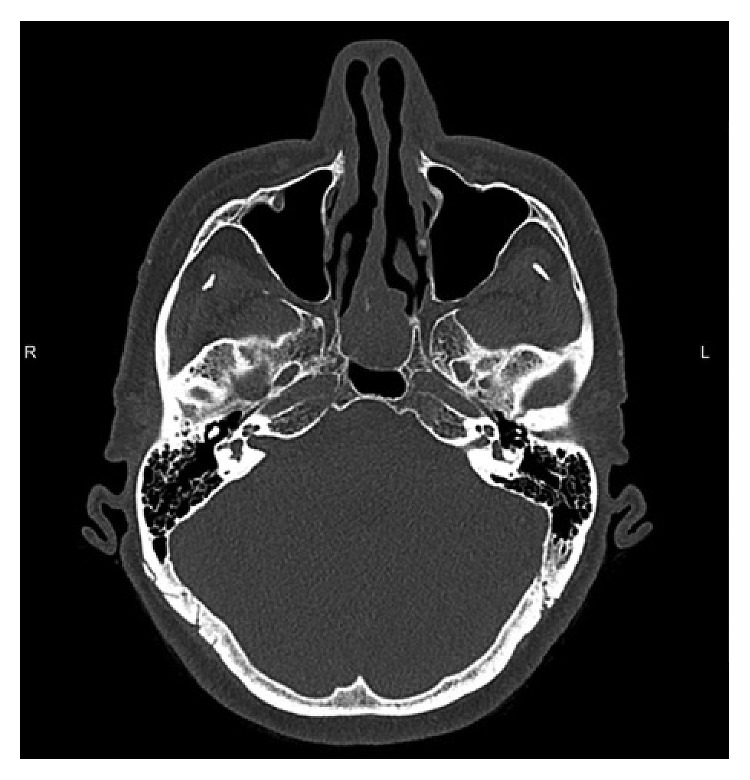
Axial CT image showing a soft tissue mass centered at the posterior nasal septum with extension into the nasopharynx.

The patient was referred to our institution for further management. Preoperative CT imaging showed a smoothly marginated, soft tissue density mass centered at the posterior nasal septum with extension into the nasopharynx and bulging into the right sphenoid sinus (Figure 1). Severe thinning and smooth remodeling of the anterior wall of the sphenoid sinus and anterior clivus were seen. MRI imaging demonstrated hyperintense signaling of the mass on T1-weighted images with a peripheral rim of hypointense signaling on T2-weighted imaging suggestive of a capsule.

**Figure 2:**
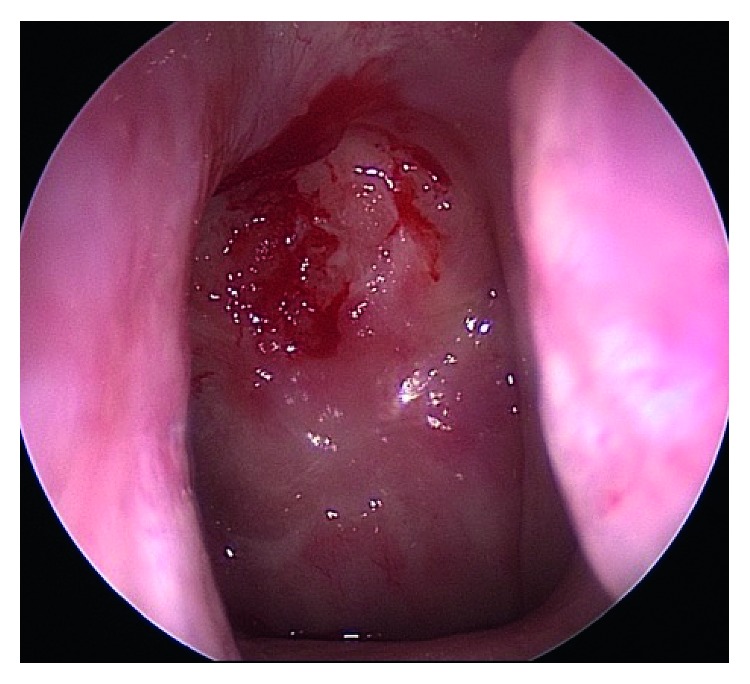
Intraoperative image of the mass protruding from the posterior septum within the left nasal cavity.

**Figure 3:**
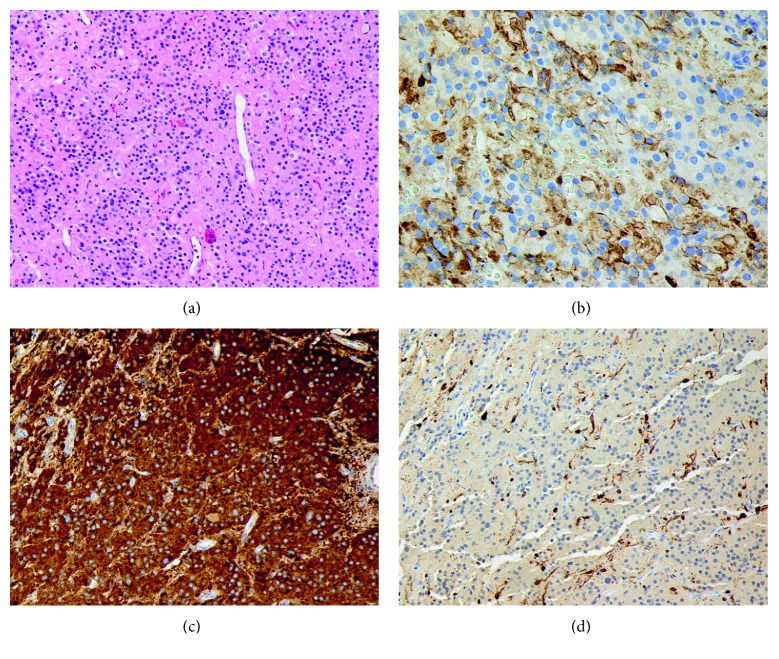
(a) H*&*E of the septal mass showing a “zellballen” pattern of clusters of epithelioid cells between bands of fibrillary stroma (20x). IHC staining showing positivity for (b) chromogranin A within epithelioid cells (40x), (c) synaptophysin within epithelioid cells (20x), and (d) S-100 within fibrillary cells (20x).

An endoscopic resection of the mass was performed. Intraoperatively, a large mass was seen in the posterior aspect of the septum, bulging into the bilateral nasal cavities and extending into the sphenoid sinuses (Figure 2). Erosion of the bone of the rostrum and anterior face of the sphenoid were also seen. Complete resection of the mass was achieved through a posterior septectomy and bilateral sphenoidotomy with tissue removal. Postoperative histologic analysis of the specimen was consistent with a paraganglioma (Figure 3).

The patient has been symptom free without local recurrence 3 months following tumor resection.

## 3. Discussion

Paragangliomas arising from the nasal cavity and nasal sinuses are extremely rare. Only 48 total cases of paragangliomas occurring in the nasal cavity or paranasal sinuses have been reported to date, occurring in patients with ages ranging between 8 and 89 years old, with an average age of 49 years old. These tumors are slightly more prevalent amongst females (60.4%). Twelve were reported to be malignant in nature, demonstrating either intracranial extension or metastasis to the cervical lymph nodes, brain, and bone. These masses were most frequently reported to originate from the ethmoid sinuses, middle turbinate, and maxillary sinuses. Other less common locations of origin reported include the superior and inferior turbinates, and the sphenoid and frontal sinuses. To the best of our knowledge, there has only been one other reported case of a paraganglioma originating from the nasal septum (Table 1) [8].

The most commonly reported symptoms included nasal obstruction, headache, and recurrent epistaxis. The vast majority of reported cases were functionally inactive, with no evidence of catecholamine or adrenocorticotropic hormone (ACTH) secretion. However, there have been a few reported cases of metabolically active tumors secreting ACTH and catecholamines, causing hypertension and other cushingoid features in the affected patient [12, 13, 30]. As such, testing for catecholamine and metanephrines is recommended in symptomatic patients [29, 33].

The origin of paragangliomas in the nasal cavity and paranasal sinuses remains unclear. Although paraganglionic tissue has never been demonstrated in the nasal cavity, a wider distribution of paraganglionic tissue is thought to exist in fetuses and neonates, degenerating after birth [42, 43]. Some authors have suggested that the migration of these embryonic paraganglionic cells accounts for the occurrence of paragangliomas in areas with no known paraganglionic tissue like the nasal cavity [13, 29, 42, 44]. Additionally, it has been shown that paraganglionic tissue exists around the terminal portion of the maxillary artery in infants and could possibly represent a point of origin for paragangliomas in the nasal cavity due to its anatomic proximity [19, 45]. Others have suggested that the paraganglionic tissue may exist in the pterygopalatine fossa due to the close relationship of paraganglionic tissue with arteries and cranial nerves [18, 19, 22].

In contrast, chondrocytes of the cartilaginous septum develop from neural crest cells between the nasal cavities by the ninth week of embryonic development [46, 47]. This is evident by the expression of various neural crest stem cell markers in nasal septum progenitor cells [48]. Several studies have demonstrated that these neural crest-derived chondrocytes in an adult septum retain pluripotent properties, with the capacity for osteogenic, chondrogenic, and neurogenic differentiation [48, 49]. It is possible that the neural crest origin of septal chondrocytes plays an important factor in the development of nasal septal paragangliomas. Despite the neural crest origins of nasal chondrocytes, paragangliomas arising from the nasal septum are extremely rare.

Masses arising from the nasal septum represent a broad spectrum of diagnoses including polyps, chondromas, hematomas, and hemangiomas. Less common septal lesions include sarcomas, melanomas, and other neoplasms [46, 50]. Patients with septal masses typically present with nonspecific symptoms of nasal obstruction or epistaxis, making it difficult to diagnose these masses based on clinical evaluation alone. CT imaging and MR imaging are useful tools in determining exact locations and boundaries of lesions, as well as identifying any erosion or extension into surrounding structures, though imaging features of most masses are nondiagnostic. Diagnosis of such masses requires a combination of clinical history and histopathologic findings. Although paragangliomas of the nasal septum are extremely rare, they should be included in the differential diagnosis when evaluating a septal mass.

The classical histologic appearance of a paraganglioma features an alveolar pattern of well-defined clusters of epithelioid chief cells with round nuclei and eosinophilic cytoplasm. These cells form a “zellballen” pattern of nests separated by vascular stroma and spindle-shaped sustentacular cells [28, 43]. Immunohistochemical staining of paragangliomas generally demonstrates positive markers for NSE, synaptophysin, and chromogranin within the chief cells and S-100 and GFAP within the sustentacular cells [8, 25, 43].

Although the majority of reported nasal paragangliomas are benign, 12 of the cases presented in this review (25.0%) were reported to be malignant, demonstrating intracranial invasion and/or metastasis to the brain, cervical lymph nodes, and bones. It is widely accepted that malignancy cannot be diagnosed by histology alone, but requires evidence of bony invasion or distant metastasis [18, 19, 22]. Certain histological features such as mitotic figures, cellular pleomorphism, necrosis, and vascular invasion can be suggestive of malignancy, though none of these features have been shown to be reliable predictors of malignancy [19, 51]. As such, evaluation with CT imaging and MRI imaging plays a key role in both localization of the primary tumor and identification of local invasion or metastasis.

Almost all of the cases presented in this review were treated surgically. Suggested management of sinonasal paragangliomas involves surgical excision with close follow-up, as several cases of recurrence have been reported [4, 22, 29]. Due to the rich vascularity of these masses, preoperative embolization of the vessels supplying the tumor has been suggested to reduce bleeding during surgery [4, 22, 24]. Many authors suggest the use of radiation therapy to slow the rate of recurrence, although radiation therapy alone has not been shown to be curative [3, 22, 33]. Chemotherapy as a primary treatment for these tumors has been shown to be largely ineffective, though 3 cases reported in this review reported the use of chemotherapy in addition to surgery and radiation therapy in the management of malignant sinonasal paragangliomas [4, 22].

## 4. Conclusion

We present a rare case of a paraganglioma presenting as a septal mass. To the best of the authors' knowledge, only one other case of nasal septal paragangliomas has been reported. Clinically, these lesions cause symptoms of obstruction, congestion, headache, and epistaxis. Recognizing that paragangliomas can arise from the nasal septum is crucial to accurately diagnose these tumors when evaluating septal lesions. A diagnosis of a paragangliomas can be confirmed based on the histopathologic imaging. These tumors are usually benign but require additional imaging as malignancy cannot reliably be ruled out on the basis of histology alone. Treatment of these tumors requires surgical resection, though radiation therapy has been to show slow growth and decrease recurrence.

## Figures and Tables

**Table 1:** Review of reported cases of sinonasal paragangliomas.

Case	Author	Year	Sex	Age	Location	Biological behavior	Therapy	Follow-up
1	Harkins [9]	1957	F	52	Ethmoid sinus	Benign	Exc, RT, lig ECA	NED at 1 year
2	Moran [10]	1962	F	89	Nasal cavity	Benign	Exc, ligation of external carotid artery	NED at 2 years
3	Lack [3]	1976	F	50	High nasal vault	Benign (local recurrence x3)	Exc, ligation of external carotid artery	NED at 11 years following final exc
4			F	50	Middle turbinate	Benign	Exc	No f/u
5			M	8	Middle turbinate	Benign	Exc	NED at 7 years
6	Gosavi [11]	1978	M	65	Middle turbinate	Benign	Exc, RT (incomplete cycle)	NED at 2 years
7	Apple [12]	1982	F	50	Ethmoid, sphenoid, and maxillary sinuses	Benign; ectopic ACTH production	Exc, RT, hormonal pharmacotherapy	NED at 6 years
8	Koegel [13]	1982	F	71	Maxillary and sphenoid sinuses	Malignant (extension into temporal lobe); catecholamine secretion	RT	No follow-up available
9	Himelfarb [14]	1985	M	41	Middle turbinate	Benign	Exc	NED at 1 year
10	Straehler [15]	1985	M	42	Maxillary sinus	Benign	Exc	No follow-up available
11	Ueda [16]	1985	F	31	Maxillary and ethmoid sinuses	Benign	Exc	NED at 2.5 years
12	Watson [17]	1988	M	56	Inferior turbinate	Benign	Exc, RT	NED at 3 years
13	Branham [18]	1989	F	25	Maxillary and ethmoid sinuses	Malignant (brain metastasis)	Exc, RT, CT	DOD at 3 years
14	Kuhn [19]	1989	M	62	Superior nasal cavity, ethmoid sinus	Benign	Exc	NED at 1 year
15	Shimoda [20]	1989	M	58	Ethmoid sinus and bilateral frontal fossa	Malignant (cervical lymph nodes metastasis)	Exc, RT	NED at 2 years
16	Talbot [21]	1990	F	17	Maxillary sinus	Benign	Embolization of internal maxillary artery, exc	NED at 3 months
17	Nguyen [22]	1995	F	32	Ethmoid sinus	Malignant (bone metastasis)	Exc, RT	DOD at 15 years
18	Biswas [23]	1999	F	45	Middle turbinate	Benign	Exc	NED at 1 month
19	Sharma [24]	1999	M	33	Frontal sinus (primary); orbit, optic nerve, cavernous sinus, maxillary and ethmoid sinuses (recurrent)	Malignant (recurrence, intracranial invasion)	CT, RT, exc; vessel embolization, exc 8 months later for recurrence	DOD at 19 months
20	Welkoborsky [25]	2000	F	54	Nasal cavity	Benign	Exc	NED at 3 years
21			F	56	Ethmoid sinus	Malignant (brain metastasis)	Exc, RT	DOD at 28 months
22			M	57	Ethmoid sinus	Benign	Exc, RT	NED at 2 years
23	Scott [26]	2001	M	24	Nasal cavity, ethmoid sinus	Benign	Exc	NED at 15 months
24	Lecuna [27]	2002	F	72	Ethmoid sinus (primary); maxillary sinus (recurrent)	Malignant (recurrence, submandibular lymph nodes metastasis)	Exc	No follow-up available
25	Ketabchi [28]	2003	F	72	Nasal cavity	Benign	Exc	NED at 8 months
26	Mouadeb [29]	2003	M	72	Ethmoid sinus	Benign	Exc	NED at 4 years
27	Lieberum [30]	2003	M	64	Frontal and ethmoid sinuses	Benign; ectopic ACTH production	Exc	NED at 2 years
28	Askar [31]	2003	M	47	Middle turbinate	Benign	Exc	NED at 2 years
29	Rocha [32]	2005	M	45	Nasal cavity	Benign	Exc	NED at 5 months
30	Liess [33]	2007	F	64	Sphenoid sinus, sella, cavernous sinus	Benign	Partial exc, RT	Residual tumor stable at 2 years
31	Morales [7]	2007	F	41	Sphenoid and ethmoid sinuses	Benign	No information available	No follow-up available
32	Jin [34]	2008	F	23	Superior turbinate	Malignant (questionable transformation simulating Ewing's sarcoma/primitive neuroectodermal tumor)	Exc	NED at 3 years
33	Fasunla [35]	2008	F	39	Lateral nasal wall	Benign	Exc	NED at 1 year
34	Kisser [6]	2012	F	36	Maxillary sinus	Benign	Exc	NED at 1 year
35	Zainine [36]	2012	F	43	Middle turbinate	Benign	Exc	NED at 2 years
36	Papaspyrou [4]	2013	F	33	Nasal cavity	Benign	Exc	NED at 15 years
37			M	64	Ethmoid sinuses, anterior skull base, frontal lobe dura (primary); ethmoid, sphenoid, cavernous sinus, orbit (recurrent)	Malignant (recurrent, intradural metastases)	2 exc, RT, CT	DOD at 60 months
38			F	56	Ethmoid sinus	Malignant (brain metastasis)	Exc, RT	DOD at 28 months
39			F	80	Nasal cavity, maxilloethmoidal angle, maxillary, ethmoid, and sphenoid sinuses (primary); nasal cavity, anterior skull base, frontal lobe (recurrent)	Malignant (recurrent, intracranial invasion)	Exc	DNOD 17 months after 2nd surgery
40			M	57	Ethmoid sinus	Benign	Exc	NED at 2 years
41			F	54	Nasal cavity	Benign	Exc	NED at 3 years
42	Granato [5]	2013	F	61	Nasal cavity	Benign	Exc	NED at 2 years
43	Michel [37]	2013	M	47	Maxillary sinus	Malignant (cervical lymph node metastasis)	Exc, RT	NED at 1 year
44	Amiraraghi [38]	2013	M	29	Cribriform plate and fovea ethmoidalis presenting as nasal polyps	Benign	Exc	NED at 3 years
45	Arora [39]	2014	F	40	Lateral nasal wall	Benign	Exc	NED at 2 years
46	Yamaguchi [40]	2015	F	66	Ethmoid sinus	Benign	Exc	NED at 1 year
47	Aydin [41]	2015	F	32	Nasal cavity, ethmoid sinus	Benign (local recurrence x2)	Exc	NED 5 years after final exc
48	Srivastava [8]	2016	M	60	Anterior nasal septum	Benign	Exc	NED at 18 months

RT, radiation therapy; CT, chemotherapy; Exc, surgical excision; NED, no evidence of disease; DOD, died of disease.
